# Phylogeography in *Nassarius* mud snails: Complex patterns in congeneric species

**DOI:** 10.1371/journal.pone.0180728

**Published:** 2017-07-12

**Authors:** Chuanliang Pu, Haitao Li, Aijia Zhu, Yiyong Chen, Yan Zhao, Aibin Zhan

**Affiliations:** 1 Research Center for Eco-Environmental Sciences, Chinese Academy of Sciences, Haidian District, Beijing, China; 2 University of Chinese Academy of Sciences, Shijingshan District, Beijing, China; 3 South China Sea Environmental Monitoring Center, State Oceanic Administration, Guangzhou, Guangdong, China; National Cheng Kung University, TAIWAN

## Abstract

One major goal for phylogeographical studies is to elucidate respective roles of multiple evolutionary and ecological forces that shape the current distribution patterns. In marine and coastal ecosystems, it has been generated a common realization that species with enormous population size and pelagic larval stages can disperse across broad geographical scales, leading to weak or even no phylogeographical structure across large geographical scales. However, the violation of such realization has been frequently reported, and it remains largely unexplored on mechanisms responsible for various phylogeographical patterns observed in different species at varied geographical scales. Here, we used a species-rich genus *Nassarius* to assess and compare phylogeographical patterns in congeneric species, and discuss causes and consequences underlying varied phylogeographical patterns. Interestingly, we observed complex phylogeographical patterns both within single species and across multiple species, and multiple analyses showed varied levels of genetic heterogeneity among sites within and across species. Available evidence suggests that related species with similar biological characteristics may not be necessary to result in consistent phylogeographical patterns. Multiple factors, including larval ecology, interactions between dispersal and natural selection, and human activity-mediated dispersal, can partially explain the complex patterns observed in this study. Deep investigations should be performed on these factors, particularly their respective roles in determining evolutionary/ecological processes to form phylogeographical patterns in species with high dispersal capacities in marine and coastal ecosystems.

## Introduction

One of the major goals of phylogeographical analyses is to elucidate relative roles of multiple evolutionary and ecological forces that shape the current distribution patterns of biodiversity [1, 2]. In summary, two major competing forces in the marine realm, that is historical vicariant (isolating) events such as geological barriers versus recent events such as recent gene flow, are commonly used to interpret the phylogeographical patterns and population genetic structure at varied geographical scales [3–6]. Widespread ancestors by vicariant/isolating events lead to disjunct distributions of genetic diversity, while recent gene flow, either by natural processes such as natural dispersal or by human-mediated gene flow, can potentially prevent both reproductive isolation and morphological divergence, resulting in weak phylogeographical patterns and strong genetic homogeneity among isolated populations [2, 3, 7].

As historical vicariance and recent gene flow do not function absolutely independently in marine and coastal ecosystems, their complex interactions have been used to interpret the current phylogeographical and population genetic patterns in many marine species [2–7]. Geological events such as the formation of bays and seaways can create physical and/or biogeographical barriers, resulting in genetic disjunctions over a long historic time scale. For example, biological evidence suggests that the formation of a mid-peninsula seaway during the late Miocene to middle Pleistocene should result in a narrow but deep mid-peninsula phylogeographical break in Baja, California, USA [8]. In addition, the population isolation due to Pleistocene sea level fluctuations was also considered as a major contributor to genetic disjunctions [8, 9]. However, as many marine species often have enormous population size and pelagic larval stages, a high dispersal capacity associated with these biological characteristics can potentially connect populations demographically across broad geographical scales, leading to weak or even no phylogeographical structure across large geographical scales [3, 4, 6, 10].

Despite that significant efforts have been made to elucidate the relative roles and contributions of multiple forces to the current phylogeographical patterns, there has been a lack of consensus so far [11]. Some species showed a high level of gene flow at large geographical scales [3, 4, 6, 10], whereas others had complex biogeographical patterns such as unstructured genetic heterogeneity at fine geographical scales [12, 13]. Such difference is mainly explained by complex interactions of unique hydrological characteristics and several biological features, such as direct versus indirect development models [13, 14], and/or the presence of lecithotrophic versus planktotrophic larvae [13–15]. For instance, a comprehensive study in 50 marine species on the Pacific coast of North America showed clear difference in the population genetic structure between species with and without pelagic development [16]. Therefore, deep investigations are largely needed to clarify possible effects of multiple factors on phylogeographical patterns in the marine realm, particularly the interactions among hydrological features, environmental factors, and biological characteristics.

Coastal sessile gastropods represent good models for elucidating the causes and consequences underlying phylogeographical patterns and geographical distributions of population genetic diversity, as they are sessile once their planktonic larvae have settled [10–16]. The passive dispersal at larval stages *via* marine currents is the major, and often the only, natural dispersal means for gene flow among populations, particularly at large geographical scales [10–16]. Consequently, planktonic larvae advected by marine currents play a significant role in the formation of phylogeographical and population genetic patterns in coastal sessile gastropods [12–16], and relatively direct evidence can be obtained by testing the interplays between planktonic larvae and marine currents based on coastal sessile gastropods, such as species with versus without planktonic larval phases [13]. As biological characteristics play crucial roles in interactions among multiple factors such as hydrological features, sister species with the same or similar biological characteristics represent promising models to test how these factors interplay to shape phylogeographical patterns.

*Nassarius*, which is a small-sized (usually < 5 cm) but species-rich (>35 recorded species) genus of Nassariinae, is widely distributed throughout worldwide oceans [17–18]. In China, it is widely distributed on inter- to sub-tidal shallow coastal regions from the north to south in all four seas (i.e., Bohai, Yellow, East and South China Seas), with a significant higher species richness along the coast of South China Sea (> 20 species) [18]. *Nassarius* snails have a scavenging-based diet, and are therefore crucial for maintaining the stability of food webs and integrity of benthonic communities [17–19]. Compared to other gastropods, *Nassarius* snails have a relatively long planktonic larval phase (1–2 months) [20], leading to a high level of dispersal capacity advected by marine currents [20, 21]. As a result, the lack of phylogeographical and population genetic differentiation is expected at relatively large geographical scales (i.e., genetic homogeneity hypothesis). In this study, we chose the species-rich genus of *Nassarius* along the Chinese coast to test the genetic homogeneity hypothesis in sister species with similar biological characteristics. In particular, we aim to investigate and compare phylogeographical patterns in multiple sister species at different geographical scales, and further to discuss potential factors responsible for observed patterns in this study.

## Materials and methods

### Sampling and species identification

**Fig 1:**
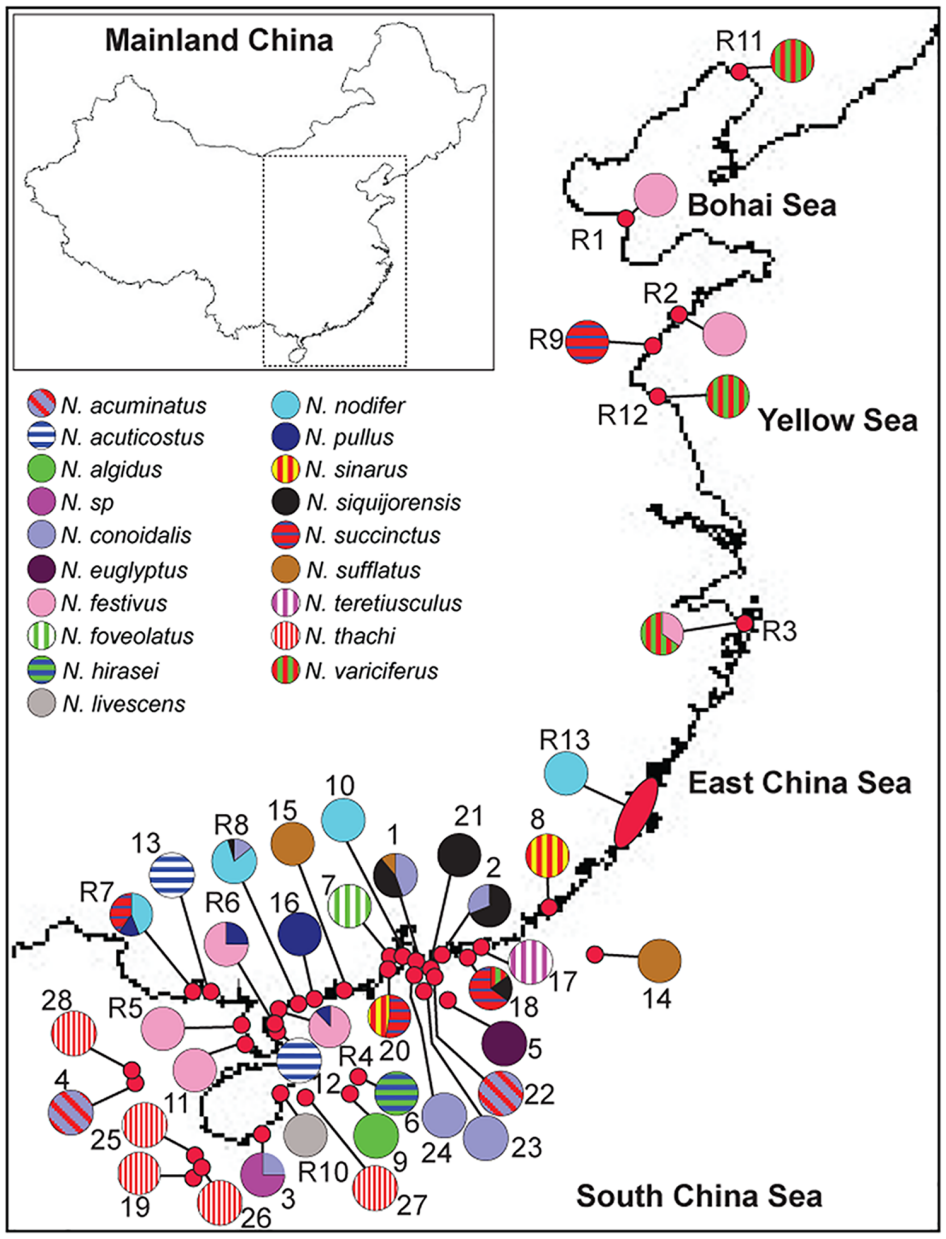
Sampling locations and species delimitation for the *Nassarius* mud snails collected from the Chinese coast. The species are color-coded as shown in the legend, and the pie chart at each location is proportional to the observed frequency of each species. The sampling site name is as per Table 1. All maps are made by ArcGIS version 10.0 (ESRI Company, USA).

Our study focuses on marine invertebrates, so we do not need permit for both research and field collection. The field studies did not involve endangered or protected species. Our sampling design aimed to maximize the geographical coverage of *Nassarius* snails along the Chinese coast, especially species-rich regions on the South China Sea (Fig 1). A total of 28 sites were surveyed to collect all *Nassarius* individuals found during field surveys. In addition, 13 sites from Zou et al. [22] were also included in this study. A total of 358 individuals collected from 41 sites were used for downstream analyses (Table 1; Fig 1).

**Table 1:** Sampling site, species delimitation, and mitochondrial cytochrome *c* oxidase subunit I (COI) and nuclear internal transcribed spacer 1 (ITS 1) diversity for the *Nassarius* mud snails. The sites coded with “R” were adopted from Zou et al. [22], and accession numbers for sequences derived from these sites were listed in S1 Table of Zou et al. [22]. *N* = number of individuals sequenced; *n* = number of haplotypes/alleles; *h* = haplotypic/allelic diversity; *π* = nucleotide diversity. For sampling sites, the abbreviations for provinces are: SD = Shandong Prov., ZJ = Zhejiang Prov., GD = Guangdong Prov., HN = Hainan Prov., LN = Liaoning Prov., JS = Jiangsu Prov., FJ = Fujian Prov., GX = Guangxi Zhuang Autonomous Region.

Site	Site Code	Species	Cytochrome *c* oxidase subunit I (COI)					Internal transcribed spacer 1 (ITS 1)				
			*N*	*n*	*h*	*π*	Haplotype code	*N*	*n*	*p*	*π*	Allele code
Kenli, SD	R1	*N*. *festivus*	10	9	0.978	0.005	C_NFe1-9	8	1	-	-	I_NFe_1
Jimo, SD	R2	*N*. *festivus*	8	6	0.929	0.033	C_NFe2, 5, 6, 10–12	6	2	0.333	0.001	I_NFe_1–2
Ningbo, ZJ	R3	*N*. *festivus*	8	8	1.000	0.049	C_NFe11, 13–18	7	3	0.524	0.001	I_NFe_2–4
		*N*. *variciferus*	15	12	0.952	0.008	C_NVa1-12	8	8	1.000	0.042	I_NVa_1–8
Caotan, GD	R4	*N*. *festivus*	7	7	1.000	0.006	C_NFe15, 19–24	6	3	0.833	0.002	I_NFe_1–3
		*N*. *pullus*	1	1	-	-	C_NPu1	1	1	-	-	I_NPu_6
Jiaowei, GD	R5	*N*. *festivus*	3	2	0.667	0.004	C_NFe15, 25	3	3	1.000	0.002	I_NFe_1–3
Xinliao, GD	R6	*N*. *festivus*	3	3	1.000	0.037	C_NFe9, 15, 26	3	3	1.000	0.004	I_NFe_1, 3, 5
		*N*. *pullus*	1	1	-	-	C_NPu2	1	1	-	-	I_NPu_5
Beihai, GX	R7	*N*. *nodifer*	14	11	0.934	0.006	C_NNo1-11	7	7	1.000	0.018	I_NNo_1–7
		*N*. *pullus*	5	4	0.900	0.004	C_NPu2-5	4	4	1.000	0.008	I_NPu_1–4
		*N*. *succinctus*	12	9	0.886	0.004	C_NSuc1-9	2	2	1.000	0.000	I_NSuc_1, 2
Zhanjiang, GD	R8	*N*. *conoidalis*	3	3	1.000	0.006	C_NCo1-3	3	3	1.000	0.011	I_NCo_1–3
		*N*. *nodifer*	17	9	0.787	0.003	C_NNo2, 10, 12–18	6	6	1.000	0.016	I_NNo_5, 8–12
		*N*. *siquijorensis*	1	1	-	-	C_NSiq1	2	2	1.000	0.013	I_NSiq_1–2
Rizhao, SD	R9	*N*. *succinctus*	7	4	0.714	0.001	C_NSuc1, 10–12	2	1	-	-	I_NSuc_1
Wenchang, HN	R10	*N*. *livescens*	4	3	0.833	0.028	C_NLi1-3	4	2	0.500	0.003	I_NLi_1–2
Panjin, LN	R11	*N*. *variciferus*	14	11	0.846	0.005	C_NVa10, 13–22	4	3	0.833	0.002	I_NVa_7, 9–10
Guanyu, JS	R12	*N*. *variciferus*	5	4	0.900	0.005	C_NVa12, 20, 23–24	1	1	-	-	I_NVa_11
Coast along FJ	R13	*N*. *nodifer*	24	24	1.000	0.005	C_NNo2, 4, 10, 12, 19–28	-	-	-	-	-
Pearl River estuary, GD N22°19.78', E113°47.97'	1	*N*. *siquijorensis*	4	3	0.833	0.031	C_NSiq2-3, 13	3	3	1.000	0.004	I_NSiq_3–5
		*N*. *conoidalis*	4	2	0.800	0.024	C_NCo4-5	4	4	1.000	0.103	I_NCo4-7
		*N*. *sufflatus*	2	2	1.000	0.002	C_NSuf2-3	2	2	1.000	0.008	I_NSuf1-2
Daya Bay, GD N22°11.93', E113°48.0'	2	*N*. *siquijorensis*	11	8	0.927	0.032	C_NSiq4-8, 15–17	5	4	0.833	0.005	I_NSiq_6–9
		*N*. *conoidalis*	5	5	1.000	0.009	C_NCo6, NCo8-11	3	3	1.000	0.091	I_NCo12-14
Lingshui Bay, HN N18°17'46.7'', E109° 51' 18.1''	3	*N*. *conoidalis*	1	1	-	-	C_NCo7	1	1	-	-	I_NCo15
		*N*. *sp*	3	2	0.067	0.019	C_Nsp1-2	2	2	1.000	0.009	I_Nsp1-2
Beibu Gulf N18°28' 47.6", E107°43' 28.2"	4	*N*. *acuminatus*	3	2	0.667	0.003	C_NAc1-2	1	1	-	-	I_NAc1
South China Sea N21°25'08'', E115° 25' 57''	5	*N*. *euglyptus*	3	3	1.000	0.006	C_NEu1-3	1	1	-	-	I_NEu1
Offshore of Hainan Prov. N19°45'01'', E112° 34' 21''	6	*N*. *hirasei*	3	2	0.667	0.012	C_NHi1-2	3	2	1.000	0.011	I_NHi1-2
Guanghai Bay, GD N21°54'46'', E112° 47' 15''	7	*N*. *foveolatus*	1	1	-	-	C_NFo1	1	1	-	-	I_NFo1
Nan'ao Island, GD N23°27'57'', E117° 1' 48''	8	*N*. *sinarus*	6	4	0.800	0.002	C_NSin1-4	6	5	0.933	0.003	I_NSin1-5
Offshore of HN N19°28'44'', E112° 10' 54''	9	*N*. *algidus*	2	2	1.000	0.014	C_NAl1-2	1	1	-	-	I_NAl1
Guishan Island, GD N22°8'36'', E113° 48' 35''	10	*N*. *nodifer*	3	2	0.667	0.003	C_NNo14, 29	2	2	1.000	0.025	I_NNo_13–14
Liusha Bay, GD N20°28'17'', E109° 52' 42''	11	*N*. *festivus*	1	1	-	-	C_NFe21	1	1	-	-	I_NFe_6
Xinliao Island, GD N20°38'44'', E110° 29' 36''	12	*N*. *acuticostus*	1	1	-	-	C_NAcu1	1	1	-	-	I_NAcu1
Beilun River Estuary, GX N21°38'16'', E108° 22' 26''	13	*N*. *acuticostus*	1	1	-	-	C_NAcu1	1	1	-	-	I_NAcu2
Offshore of GD N21°39'18', E118° 48' 35''	14	*N*. *sufflatus*	1	1	-	-	C_NSuf1	1	1	-	-	I_NSuf3
Yangjiang, GD N21°45'20'', E112° 3' 30''	15	*N*. *sufflatus*	6	5	0.933	0.007	C_NSuf2-6	1	1	-	-	I_NSuf2
Maoming, GD N21°30'34'', E111° 3' 27''	16	*N*. *pullus*	3	3	1.000	0.002	C_NPu2, 4, 6	2	2	1.000	0.011	I_NPu_7–8
Shanwei, GD N22°46'47'', E115° 23' 22''	17	*N*. *teretiusculus*	1	1	-	-	C_NTe1	1	1	-	-	I_NTe1
Honghai Bay, GD N22°39.12', E115°2.35'	18	*N*. *variciferus*	10	8	0.911	0.008	C_NVa5, 10, 13, 25–29	10	10	1.000	0.008	I_NVa6, 12–20
		*N*. *siquijorensis*	4	4	1.000	0.039	C_NSiq1, 9–10, 14	1	1	-	-	I_NSiq_1
		*N*. *succinctus*	13	9	0.936	0.007	C_NSuc1-3, 5, 13, 21–24	14	11	0.835	0.004	I_NSuc_1, 3–12
Offshore of Sanya, HN N17°37.5', E108°18.58'	19	*N*. *thachi*	3	3	1.000	0.002	C_NTh1-3	1	1	-	-	I_NTha1
Taishan, GD N21°48.98', E112°53.23'	20	*N*. *succinctus*	22	15	0.896	0.008	C_NSuc1-2, 4–5, 10, 13–20, 25–26	20	12	0.589	0.002	I_NSuc_1, 5, 12–21
		*N*. *sinarus*	20	15	0.916	0.004	C_NSin1, 5–18	20	11	0.711	0.002	I_NSin5-15
Offshore of Hongkong Island N22°17'41'', E114°11' 40''	21	*N*. *siquijorensis*	3	3	1.000	0.005	C_NSiq5, 11–12	1	1	-	-	I_NSiq_10
South of Erzhou Island, GD N21°54'49", E114°15'58"	22	*N*. *acuminatus*	8	4	0.750	0.006	C_NAc3-6	3	3	1.000	0.005	I_NAc2-4
South of Erzhou Island, GD N21°16' 21", E113°53'34"	23	*N*. *conoidalis*	7	2	0.571	0.001	C_NCo18-19	2	2	1.000	0.003	I_NCo16-17
South of Erzhou Island, GD N21°51' 00", E113°40' 30"	24	*N*. *conoidalis*	6	6	1.000	0.008	C_NCo12-17	4	4	1.000	0.065	I_NCo8-11
South of Erzhou Island, GD N17°50' 33", E108°17'56"	25	*N*. *thachi*	2	2	1.000	0.007	C_NTh4-5	2	2	1.000	0.004	I_NTh2-3
South of Erzhou Island, GD N17°34.46', E108°24.663'	26	*N*. *thachi*	2	2	1.000	0.012	C_NTh6-7	1	1	-	-	I_NTh4
South of Erzhou Island, GD N19°10.000', E111°00.000	27	*N*. *thachi*	2	1	-	-	C_NTh6	1	1	-	-	I_NTh4
South of Erzhou Island, GD N18°30' 46", E107°38' 20"	28	*N*. *thachi*	1	1	-	-	C_NTh6	1	1	-	-	I_NTh4
Total	41 sites		330	204	-	-	-	202	134	-	-	-

All collected individuals were identified based on both shell morphology and characteristics of radular teeth. For the radular teeth observation, the radular sacs were removed from the soft part and dissolved in 10% sodium hydroxide until the radular teeth were completely isolated from soft tissues. The teeth were cleaned in double distilled water for three times and stained with 5% chromic acid for 5 min. Subsequently, the prepared teeth were mounted on glass slides and observed under a light microscope (OLYMPUS BX51, Japan).

### DNA extraction, PCR amplification and sequencing

Based on results from our former study [19] and Zou et al. [22], we chose one mitochondrial gene, the cytochrome *c* oxidase subunit I (COI), and one nuclear fragment, the internal transcribed spacer 1 (ITS1), for large-scale analyses in this study. The total genomic DNA was extracted from foot muscle tissues using the standard phenol/chloroform method. The quantity and quality of extracted DNA were measured by NanoDrop^™^ 1000 spectrophotometer. The COI and ITS1 fragments were amplified using the universal primers LCO1490-HCO2198 [23] and the primer pair ITS-1F [24] and ITS-1R [25], respectively. In addition, we designed *Nassarius*-specific COI primers (NassariusF: ACGGCHTTRAGNCTYYTWATTCGTGC; NassariusR: GTRATAGCYCCWGCTARNACNGG) based on available sequences in GenBank and those obtained in this study for the amplification of failed species and/or individuals. Polymerase chain reaction (PCR) amplifications were carried out in a total volume of 25 μL, containing 100 ng of genomic DNA, 1 x PCR buffer, 2 mM of Mg^2+^, 0.2 mM of dNTPs, 0.4 μM of each primer, and 2 U of *Taq* DNA polymerase (LA *Taq*, Takara Inc., Japan). PCR program included an initial denaturation step at 95°C for 5 min, followed by 30 amplification cycles of 95°C for 30 sec, 50°C for 30 sec, 72°C for 90 sec, and a final elongation step at 72°C for 10 min. For all COI amplicons, sequencing was directly performed using the forward primer and BigDye Terminator 3.1 sequencing chemistry on an ABI 3730 automated sequencer. For ITS1, PCR products were sequenced first and those containing inserts/deletions and/or ambiguous nucleotides were cloned using a pGEM T-easy vector system II (Promega). For each individual, eight positive clones were randomly selected for sequencing using the forward primer. Both COI and ITS1 sequences that contained ambiguous nucleotides were subsequently re-sequenced with the reverse primers.

### Data analyses

Sequences were aligned and edited using Codon-Code Aligner version 2.0.6 (CodonCode Corporation, Dedham, MA, USA). DnaSP version 5 [26] was used to identify haplotypes/alleles for both genetic markers. Bayesian inference (BI) and neighbour-joining (NJ) phylogenetic analyses were conducted based on recovered haplotypes/alleles using *Cyclope neritea* and *Pleuroploca filamentosa* as outgroups for COI and ITS1, respectively. The best DNA substitution model for each DNA fragment was determined using MrModeltest version 3.7 [27] with Akaike Information Criteria. The Bayesian analysis was conducted using MrBayes version 3.2 [28]. Trees were sampled every 100 generations for 20 million generations, and the first 25% of all the trees sampled before convergence were discarded as burn-in. NJ phylogenetic analyses were performed using MEGA version 7.0 [29] based on nucleotide distances corrected using the Tamura-Nei model. Clade supports were estimated using the bootstrap analysis with 1000 replicates. In addition, the maximum likelihood (ML) approach was also used to reconstruct phylogenies. ML phylogenies were estimated with RAxML version 8.2.10 [30] on CIPRES [31]. DNA sequences for each gene were partitioned with separate substitution models estimated using MEGA version 7.0. Rapid bootstrap analyses [32] were carried out with 500 replicates. Alignment gaps in ITS1 sequences were treated as a fifth character-state in BI phylogeny reconstruction and missing data for both NJ and ML phylogeny reconstruction.

For each species, we used DnaSP version 5 to calculate the number of haplotypes/allele (*N*_h_), haplotype/allele diversity (*h*) and nucleotide diversity (*p*), and to test whether the sequences evolved under neutrality according to Tajima’s *D* and Fu’s *F*_s_. For the six widely distributed species (*N*. *conoidalis*, *N*. *succinctus*, *N*. *festivus*, *N*. *thachi*, *N*. *siquijorensis*, *N*. *variciferus*; Fig 1), their haplotypes/alleles were mapped back to sampling sites where individuals were collected, and site-specific and commonly shared haplotypes/alleles were determined based on the geographical distributions of haplotypes/alleles for each species. For these six species, relationships among haplotypes/alleles were further examined using the median-joining network method (MJN) implemented in NETWORK [33].

To illustrate dispersal patterns, isolation-by-distance (IBD) was examined by testing the correlation between genetic distance *Φ*_ST_ and geographical distance using the Mantel test with 10000 permutations implemented in GENEPOP online version (http://www.genepop.curtin.edu.au/). We performed IBD analyses for the six widely distributed species. We measured the geographical distance between sites using the shortest coastal line with GOOGLE EARTH version 5. To detect hierarchical genetic structure among sampling sites, we performed an analysis of molecular variance (AMOVA) using ARLEQUIN version 3.5 [34] in two species (*N*. *festivus* and *N*. *variciferus*), as these two species were distributed in more than two seas. We divided all samples into four groups based on different seas: Bohai Sea, Yellow Sea, East China Sea, and South China Sea. Molecular variance was partitioned into three levels: between groups, among populations within groups, and among individuals within populations.

## Results

### Species identification based on morphology

**Fig 2:**
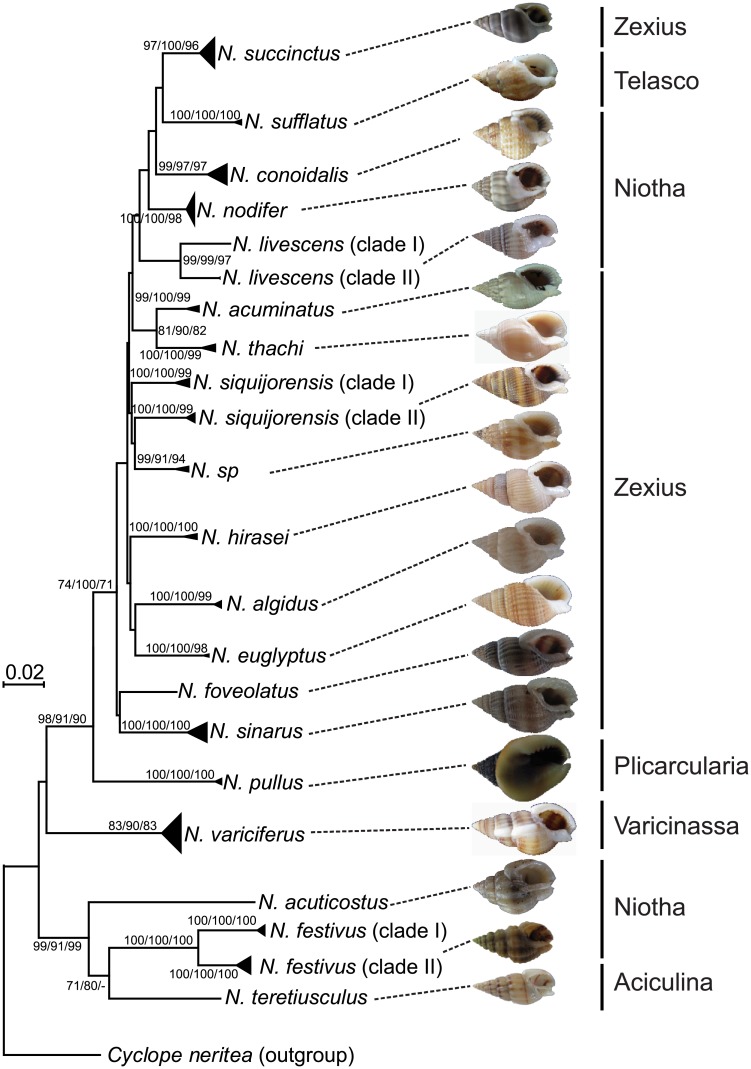
The shell morphology and neighbour-joining (NJ) tree based on the cytochrome *c* oxidase subunit 1 gene (COI) in *Nassarius* species. The commonly used sub-genus are shown on the right, and bootstrap values for NJ and maximum likelihood (ML), and posterior probabilities (in percentage) for Bayesian Inferences (BI) are shown at each major node. The values greater than 70 are shown.

After morphological analyses of taxonomic keys on both shells (Fig 2) and radular teeth (S1 Fig), we identified a total of 19 species (Table 1, Fig 1 and S1 Table). These identified species belonged to six commonly used subgenus of *Nassarius*–*Aciculina* (1 species), *Niotha* (5 species), *Plicarcularia* (1 species), *Varicinassa* (1 species), *Telasco* (1 species) and *Zexius* (10 species) (Fig 2), although such taxonomic delimitation on subgenus is not valid (see the Discussion section for more details). At most sampling sites, only one species was recovered, while the co-occurrence of multiple species was detected at 10 sampling sites (Fig 1). Only four species were identified from the Bohai, Yellow and East China Seas, while a significantly higher level of species richness (18 species) was recovered from the biodiversity hotspot, the South China Sea (Fig 1).

### COI-based phylogenetic analyses

**Fig 3:**
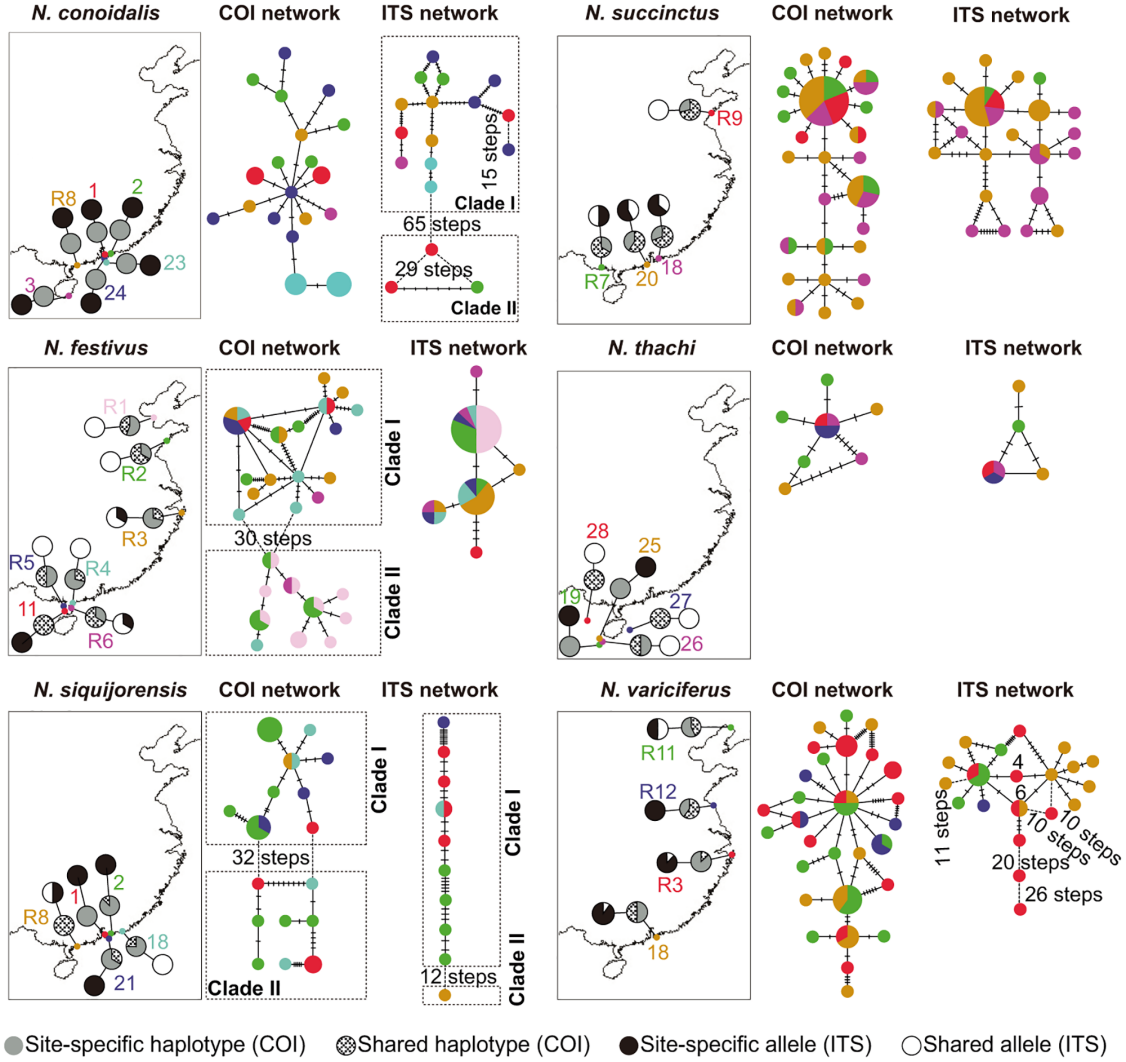
Geographical distribution of haplotypes (COI)/alleles (ITS1) and haplotype/allele networks for the six widely distributed *Nassarius* species. The pie charts on maps indicate the frequency of site-specific and shared halotypes/alleles among sampling sites. For networks, sampled haplotypes/alleles are indicated by circles and missing or unsampled are indicated by dashes. Haplotypes/alleles are color-coded according to sampling locations as shown in maps. Circle size in networks is proportional to the observed haplotype/allele frequency. All maps are made by ArcGIS version 10.0 (ESRI Company, USA).

A total of 330 individuals (92.2%) were successfully amplified and sequenced. After sequence alignment and haplotype analysis, a total of 204 haplotyples were identified (GenBank Accession Nos.: KY100528-KY100731). Both neutral evolution tests, including Tajima’s *D* and Fu’s *F*_s_, resulted in non-significant values, suggesting that COI was not under selection in all tested species. The three phylogenetic reconstruction methods, including BI, NJ and ML, revealed largely the same topology at major nodes (only NJ phylogeny shown in Fig 2). Well supported clades were clearly defined by the three phylogenetic reconstruction methods (Fig 2), and haplotype networks provided further resolution for each major clade (Fig 3). Collectively, both phylogenetic analyses and haplotype networks revealed several solid patterns. Firstly, all morphologically identified species were well supported by the phylogenetic analyses based on COI (Fig 2). However, haplotypes derived from three morphologically identified species (*N*. *livescens*, *N*. *siquijorensis*, *N*. *festivus*) did not form single clades, with two clades for each species (Fig 2), suggesting that these species remain species complex. In these three species complexes, the inter-clade genetic divergence was significantly greater than that of intra-clades. For example, the genetic divergence between the two clades of *N*. *festivus* was as large as 7.2%, greater than intra-clade divergence of 1.2% and 2.2% (Table 2). The well-supported clades were also evidenced by haplotype networks (Fig 3). For example, the two clades of *N*. *festivus* were separated by 30 mutation steps, while only several mutation steps were detected at the intra-clade level (Fig 3). Secondly, phylogenetic analyses did not support the monophyly of the six commonly used subgenera detected in this study, and species from these subgenera were mixed together in the reconstructed phylogenies (Fig 2). Thirdly, haplotypes were not grouped into sub-clades or sub-clusters based on their geographical origins in both COI phylogenies and haplotype network analyses. Haplotypes derived from the four seas were clustered without any significant pattern (Figs 2 and 3). The lack of geographical patterns was more obviously detected in haplotype network analyses, where haplotypes derived from distant geographical sites were often clustered together while those derived from neighboring locations, even from the same sites, were far separated (Fig 3). This was also detected in the three species complexes, where haplotypes from the same sampling locations were grouped into two distinct clades or clusters (Figs 2 and 3). For example, half of the *N*. *siquijorensis* haplotypes derived from the sampling location 2 was grouped into the clade I, while the other half formed the clade II (Figs 2 and 3).

**Table 2:** Number of alleles/haplotypes and ranges of pairwise differences (in percentage) of all *Nassarius* species (clades) across 41 sampling sites along the Chinese coast. *n* = number of haplotypes (COI)/alleles (ITS1); *p* = pairwise difference between haplotypes (COI)/alleles (ITS1).

Species—clade	Cytochrome *c* oxidase subunit I (COI)		Internal transcribed spacer 1 (ITS 1)	
	*n*	*p*	*n*	*p*
*N*. *acuminatus*	6	0.4–1.3%	4	0.2–0.9%
*N*. *acuticostus*	1	-	2	0.5%
*N*. *algidus*	2	1.4%	1	-
*N*. *sp*	2	1.9%	2	0.2%
*N*. *conoidalis*—clade I	-	-	14	0.2–4.8%
*N*. *conoidalis*—clade II	-	-	3	0.4–5.2%
*N*. *conoidalis*—all	19	0.2–2.2%	17	0.2–15.0%
*N*. *euglyptus*	3	0.8–1.5%	1	-
*N*. *festivus*—clade I	10	0.2–1.2%	-	-
*N*. *festivus*—clade II	16	0.2–2.2%	-	-
*N*. *festivus*—all	26	0.2–7.2%	6	0.2–0.9%
*N*. *foveolatus*	1	-	1	-
*N*. *hirasei*	2	1.9%	2	1.1%
*N*. *livescens*—clade I	1	-	-	-
*N*. *livescens*—clade II	2	0.2%	-	-
*N*. *livescens*—all	3	0.2–4.5%	2	0.7%
*N*. *nodifer*	29	0.2–1.4%	14	0.2–3.8%
*N*. *pullus*	6	0.2–0.8%	8	0.2–1.7%
*N*. *sinarus*	18	0.2–1.1%	15	0.2–0.9%
*N*. *siquijorensis*—clade I	8	0.2–2.0%	9	0.2–0.9%
*N*. *siquijorensis*—clade II	9	0.2–1.1%	1	-
*N*. *siquijorensis*—all	17	0.2–6.4%	10	0.2–1.5%
*N*. *succinctus*	26	0.2–1.6%	21	0.2–1.1%
*N*. *sufflatus*	6	0.2–1.5%	3	0.3–0.8%
*N*. *teretiusculus*	1	-	1	-
*N*. *thachi*	7	0.2–1.5%	4	0.2–0.5%
*N*. *variciferus*	29	0.2–2.9%	20	0.2–10.2%
Total	204	-	134	-

### ITS1-based phylogenetic analyses and inconsistence between COI and ITS1

**Fig 4:**
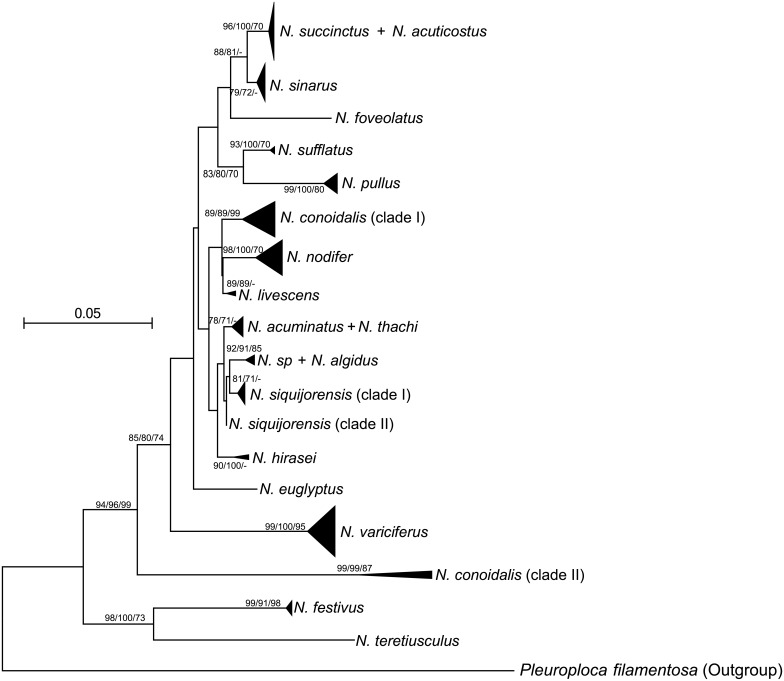
Neighbour-joining (NJ) tree based on the nuclear internal transcribed spacer 1 (ITS 1). Bootstrap values for NJ and maximum likelihood (ML), and posterior probabilities (in percentage) for Bayesian Inferences (BI) are shown at each major node. The values greater than 70 are shown.

Based on the phylogenetic analyses using COI, a total of 224 individuals were selected and amplified using the nuclear ITS1-specific primers, 202 (90.2%) of which were successful. After sequence alignment and haplotype analyses, a total of 134 alleles were identified (GenBank Accession Nos.: KY100732-KY100865; Table 1). Both neutral evolution tests, including Tajima’s *D* and Fu’s *F*_s_, showed non-significant values, suggesting that ITS1 was under neutral evolution in all tested species. Similarly to COI-based phylogeny reconstruction, the three methods (i.e., BI, NJ and ML) revealed the same topology (only NJ phylogeny shown in Fig 4). In general, ITS1 and COI largely recovered similar patterns, such as individuals from the morphologically identified species were not mixed together, phylogenetic analyses based on ITS1 did not support the monophyly of subgenus, and ITS alleles were not grouped together based on their geographical origins (Fig 4).

However, when compared to the patterns recovered by COI, some inconsistent patterns were detected based on ITS1. ITS1 generally showed a relatively lower level of resolution in phylogenetic analyses for some taxa. Several well-supported clades by both COI and morphological analyses were not well recovered in ITS1-based phylogenies, such as for *N*. *succinctus*, *N*. *acuticostus*, *N*. *acuminatus* and *N*. *thachi* (Figs 2 and 4). For the three species complexes detected based on COI, only the *N*. *siquijorensis* complex was consistently recovered by both COI and ITS1 (Fig 4), while alleles from the other two species (i.e. *N*. *livescens* and *N*. *festivus*) were grouped into single clades with low intra-clade genetic divergence (0.7% for *N*. *livescens* and 0.2–0.9% for *N*. *festivus*; Table 2). For *N*. *conoidalis*, only one clade was recovered based on COI with the haplotype genetic divergence of 0.2–2.2%; however, two well-supported clades were detected based on ITS1 with the inter-clade genetic divergence of as large as 15.0% (Fig 4; Table 2). Interestingly, these two clades were far separated by other species in the ITS1-based phylogenies (Fig 4). In addition, some clades defined by ITS1 had a relatively large range of genetic divergence, such as 0.2–4.8% for the clade I of *N*. *conoidali*s, 0.4–5.2% for the clade II of *N*. *conoidalis*, and 0.2–3.8% for *N*. *nodifer*. For *N*. *variciferus*, the intra-clade genetic divergence ranged from 0.2% to 10.2% based on ITS1, and high genetic variation among alleles was also evidenced by the network analysis, with mutation steps as large as 26 (Fig 3). In contrast, the haplotype divergence based on COI was relatively low for this species (0.2–2.9%; Table 2), and less than 10 mutation steps were detected in the haplotype network analysis (Fig 3). Often, individual members for clades defined by COI and ITS1 were not consistent. When haplotypes and alleles were examined based on individuals where they were derived from, individuals from the same clades based on COI were assigned into different clades defined by ITS1, and vice versa (Fig 3). For example, individuals of *N*. *siquijorensis* from both COI clades formed the same ITS1 clade (clade I; Fig 3). In addition, ITS1 alleles from the same individuals were often clustered into different clades while COI haplotypes were clustered closely with those from other individuals, and vice versa. All these results suggest that introgression among species occur in these individuals.

### Geographical distributions of genetic diversity

When COI haplotypes and ITS1 alleles were mapped based on their geographical origins, we found that the distributions of haplotypes/alleles were geographical distance independent. In general, haplotypes/alleles were highly site-specific. Only several haplotypes/alleles were shared among sampling sites, while a large number of haplotypes/alleles were site-specific (Fig 3). The shared haplotypes/alleles were observed in both high frequency alleles and low frequency alleles (Fig 3). In addition, haplotypes/alleles were shared among sampling sites with geographical distance of >2000km, while interestingly site-specific haplotypes/alleles were often detected in neighbouring sites with only several kilometers apart. For example, all detected COI haplotypes and ITS1 alleles were site-specific in *N*. *conoidalis* populations collected from sites with geographical distance ranging from 2 to 50km (Fig 3). Moreover, the geographical distributions of haplotypes/alleles were highly species-specific. Some species showed a high level of shared haplotypes/alleles at large geographical scales, for example, approximately 50% of the observed haplotypes/alleles were detected in *N*. *succinctus* populations collected in more than 2000 km (Fig 3). While, a high level of site-specific haplotypes/alleles was detected in some species at both fine and large geographical scales, such as in *N*. *conoidalis* and *N*. *siquijorensis* (Fig 3). Some clades were constrained in several sampling sites, for examples, the ITS1 clade II of *N*. *conoidalis* was formed by alleles derived from sampling sites 1 and 2, and the COI clade II of *N*. *festivus* was mainly restricted into the North China (sampling sites R1 and R2).

**Fig 5:**
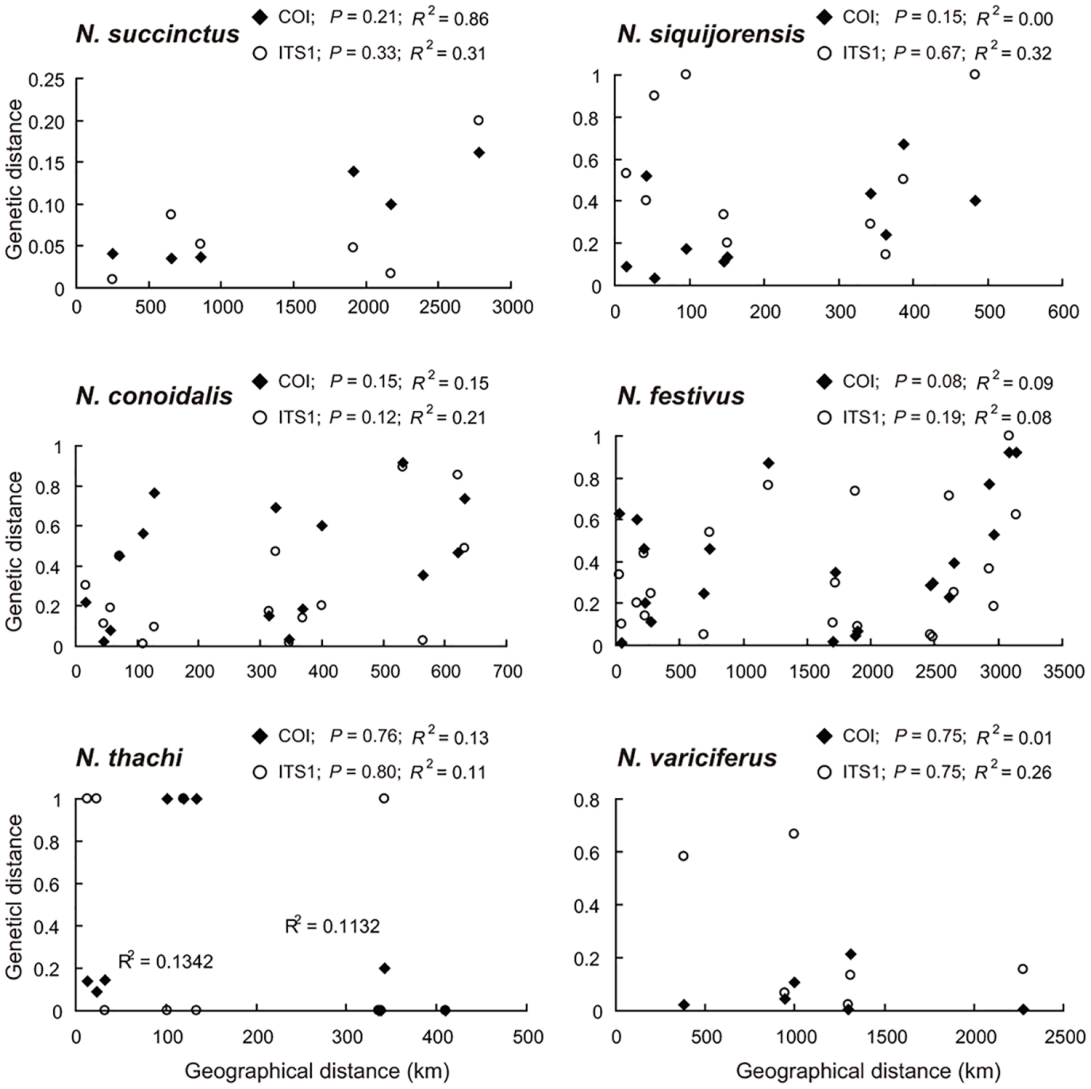
Isolation by distance (IBD) tests in the six widely distributed *Nassarius* species.

The lack of geographical pattern was also evidenced by both IBD (Fig 5) and AMOVA (Table 3). When IBD was performed in the six widely distributed species (*N*. *conoidalis*, *N*. *succinctus*, *N*. *festivus*, *N*. *thachi*, *N*. *siquijorensis*, *N*. *variciferus*; Fig 1), we observed no correlation between genetic and geographical distances for all species (Fig 5), suggesting that IBD was not characteristics of the six *Nassarius* mud snail species along the Chinese coast. This finding was further confirmed by AMOVA in two species (i.e. *N*. *festivus* and *N*. *variciferus*), where no significant among-group variation was detected (Table 3).

**Table 3:** Analysis of molecular variance (AMOVA) results based on both mitochondrial cytochrome *c* oxidase subunit I (COI) and nuclear internal transcribed spacer 1 (ITS1) for two *Nassarius* species along the Chinese coast. Sampling sites were assigned to groups based on geographical locations, i.e. Bohai Sea, Yellow Sea, East China Sea and South China Sea. **: *P* < 0.01.

Source of variation	Degree of freedom	Sum of square	Variance components	% variation	*F*-statistic
***N*. *festivus*—COI**					
Among groups	3	214.08	7.10	58.76	0.5876
Among sites within groups	3	12.07	-0.47	-3.88	0.5488
Within sites	33	179.88	5.45	45.12	-0.094**
Total	39	406.03	12.08		
***N*. *festivus*—ITS1**					
Among groups	3	4.43	-0.09	-18.18	-0.1818
Among sites within groups	3	3.56	0.30	58.91	0.4073
Within sites	27	8.21	0.30	29.27	0.4985**
Total	33	16.21	0.51		
***N*. *succinctus*—COI**					
Between groups	1	3.26	0.18	11.98	0.1198
Among sites within groups	2	1.43	-0.05	-2.96	0.0903**
Within sites	50	69.78	1.40	90.97	-0.0336
Total	53	74.46	1.53		
***N*. *succinctus*—ITS1**					
Between groups	1	0.10	-0.15	-25.97	-0.2597
Among sites within groups	2	0.85	-0.04	-6.55	-0.3252
Within sites	30	22.52	0.75	132.51	-0.0520
Total	33	23.47	0.57		

## Discussion

In this study, we used both mitochondrial COI and nuclear ITS1 to investigate and compare phylogeographical patterns of *Nassarius* mud snails along the Chinese coast, particularly in the species-rich regions on the coast of South China Sea. In contrast to the expected “genetic homogenization hypothesis”, we observed complex phylogeographical patterns, with varied levels of genetic heterogeneity within and among species at different geographical scales. Overall, we did not observe the pattern of genetic homogenization even at fine geographical scales where genetic exchanges are expected to facilitate genetic homogenization among sites owing to a long planktonic larval phase (1–2 months for *Nassarius* species [20]). Multiple analyses showed a high level of genetic heterogeneity at the haplotype/allele, population, and species levels. At the haplotype/allele and population levels, we observed a large proportion of site-specific haplotypes/alleles in populations collected even from fine geographical scales (Table 1; Fig 3). At the species level, the geographical distributions of haplotypes/alleles largely varied among species (Table 1; Fig 3), suggesting that related species with similar biological characteristics may not be necessary to result in consistent phylogeographical patterns. All these results suggest that multiple factors, as well as their complex interactions, are responsible for the observed patterns. Therefore, deep investigations should be performed on species-specific biological features, particularly interactions between species-specific biological features such as larval biology and local environments, and the roles of such interactions in determining phylogeographical patterns and evolutionary/ecological processes in species with high dispersal capacities.

Usually, marine organisms with a relatively long planktonic larval phase are expected to show weak phylogeographical structure and low population genetic differentiation, and a realization has been generalized to suggest that long planktonic durations result in a high level of dispersal [6–7, 12–15]. The extended planktonic phrases provide great opportunities for larvae/juveniles to be advected by marine currents to disperse to a wide geographical range [35], leading to spatial homogeneity along a large geographical scale (i.e., genetic homogeneity hypothesis). The genetic homogeneity hypothesis has been confirmed in many marine species with a relatively long planktonic larval phase, including bivalves and gastropods [13–14, 16]. However, surveys on multiple species showed a poor prediction on effects of planktonic larval durations on phylogeographical patterns and population genetic structure, indicating that high dispersal capacity was not necessary to result in high gene flow among populations [16]. For *Nassarius* mud snails, the planktonic larval phase of 1–2 months is expected to lead to a realized average dispersal distance ~200km/year based on the relationship between propagule duration and dispersal distance observed in other marine benthonic invertebrates [35]. In contrast to the expectation, our multiple analyses here showed complex patterns in single species and across multiple species. We observed a high level of genetic heterogeneity among majority sampling sites at both fine and large geographical scales, such as those in *N*. *conoidalis*, *N*. *siquijorensis* and *N*. *variciferus* (Fig 4). Interestingly, such genetic heterogeneity pattern was even observed at <10 km (Fig 4). However, such a heterogeneity pattern was not consistently detected in all sampling sites for a single species, such as among sites 11, R4, R5 and R6 in *N*. *festivus*, and among sites 19, 25 and 26 in *N*. *thachi*, where some haplotypes/alleles were shared among sites but some remained strictly site-specific (Fig 3). Similarly, different levels of genetic heterogeneity were detected among species, with 100% site-specific haplotypes/alleles in all sites for *N*. *conoidalis* but varied proportions among sites in other species (Fig 3). All these results suggest that the large number of site-specific haplotypes/alleles is not completely due to the small number of individuals per species at some sampling locations. The mixed results on phylogeographical structure and population genetic differentiation suggest that natural dispersal can occur to a certain geographical extent to cause gene flow, but additional powers have largely restricted genetic exchanges in the face of high dispersal in *Nassarius* species. Indeed, the mixed phylogeographical structure and genetic differentiation at varied geographical scales were detected not only in coastal molluscs [16] but also in freshwater species in flowing rivers such as the golden mussel *Limnoperna fortunei* [36] and quagga mussel *Dreissena bugensis* [37]. The extended planktonic larval stage (e.g. ~ 20 days for *L*. *fortunei*) in these mussel species in running water systems did not result in genetic homogeneity along river courses [36]. Consequently, deep investigations are largely needed to understand what additional powers can erase the effects of high dispersal to finally determine the phylogeographical patterns and population genetic structure.

Collectively, several candidate mechanisms, including larval ecology, genetic selection, habitat condition and colonization history, may explain the varied genetic differentiation in species with a long planktonic larval phrase at different geographical scales [36, 38–39]. Larval ecology, such as settlement behavior, was found to be responsible for the high genetic differentiation in marine species [40–42]. For example, direct evidence has been obtained in the *Sebastes* rockfish where different settlement behaviors resulted in varied scales of population differentiation [43]. Multiple studies hypothesized that marine sessile molluscs differed from fishes and pelagic invertebrates in which molluscs tended to form discrete populations with relatively unique community dynamics [10, 44–45]. Larval retention close to source populations was suggested to explain restricted gene flow at fine geographical scales [46]. As larval behaviors are largely species-specific and microhabitat requirements can be largely different in related species, these factors can explain the inconsistent patterns both at different geographical scales and among related species [16]. Similar to many gastropod species, there is little information available on larval ecology and habitat requirements in *Nassarius* mud snails, particularly some crucial processes such as metamorphosis and larval recruitment in the wild. As larval ecology may play a crucial role in determining many ecological and evolutionary processes, the integration of larval ecology and oceanographic factors is likely to increase the explanatory power of phylogeographical patterns and population genetic structure [12, 16, 36].

In addition to the larval ecology, natural selection associated with local environmental factors can generate substantial genetic differentiation under the circumstance of high dispersal [47–49]. For example, the salinity-associated selection of *Lap* allozyme alleles was detected in an estuarine mussel *Mytilus edulis* [47]. However, the selection alone cannot explain the patterns observed in this study, as we used relatively neutral markers classified by both Tajima’s *D* and Fu’s *F*_s_ tests. In addition, it is unusual that strong selection forces could act across species at such a fine geographical scale (<5km) in coastal ecosystems. Indeed, environmental gradients that may lead to varied selection forces rarely exist at such a fine geographical scale in coastal ecosystems, and we did not find obvious environmental gradients and/or different types of habitats at such fine scales during field sampling. Usually, selection caused by local environmental factors in coastal ecosystems is largely associated with habitat conditions such as significant environmental gradients along littoral zones. Consequently, selection associated with habitat conditions may cause genetic differentiation [47–51]. Previous studies, particularly those focusing on functional genes, have obtained direct evidence on the power of selection to generate significant structure along environmental gradients [50–51]. For example, strong selection owing to intertidal exposure resulted in significant variation in allele frequencies in the barnacle *Semibalanus balanoides* [51]. As we did not find continuous distribution of any species tested in this study along a significant environmental gradient at a relatively fine scale in our surveyed areas, we could not test the selection hypothesis associated with genetic adaptation to local habitat conditions discussed here. However, when we compared within and among species collected from different water depth such as *N*. *thachi* (86–109 meters) versus *N*. *siquijorensis* (<30 meters), we did not detect any obvious pattern and/or trend. Collectively, a balance between dispersal and selection may partially explain the variation of alleles and their frequencies detected here. Similar patterns were also detected in other species, such as barnacles [48–49].

In addition to a high level of genetic heterogeneity, genetic exchange was also observed at large geographical scales in some species such as *N*. *festivus*, with haplotypes/alleles shared among distant sites even those from different seas (Fig 4). For genetic surveys at large geographical scales, colonization history should be considered when interpreting the observed results [21, 36, 38–39]. For example, when analyzing the phylogeographical patterns of *N*. *reticulatus*, the lack of population genetic structure detected along the 1700 km northeast Atlantic coast was due to recent colonization events [52]. The genetic exchanges observed at large geographical scales in this study was likely mediated by recent human activities, as natural dispersal is impossible between many sampling sites such as those from the Bohai Sea and South China Sea. In the past three decades, human activities along the Chinese coast have been largely increasing, such as shipping and aquaculture [53]. Human activity-mediated gene flow can largely influence population genetic structure at regional, even continental scales in many marine species [38, 54].

When phylogeographical structure was assessed by two types of markers (i.e. mitochondrial COI versus nuclear ITS1), we found substantial differences between these two types of markers (Figs 2 and 4). In general, ITS1 showed a relatively lower level of resolution in phylogenetic analyses in some species, such as *N*. *livescens* and *N*. *festivus* where species complexes were detected based on COI but not based on ITS1, and *N*. *sucinctus* and *N*. *acuticostus* where these two species were well discriminated based on COI but a shallow clade was formed by these two species based on ITS1 (Figs 2 and 4). Such difference is mainly derived from the varied evolutionary rates of different genes, and the maintenance of ancestral polymorphism is expected in recently divergent species when using genes with low evolutionary rates for phylogeny reconstruction [55]. Interestingly, only one relatively shallow clade (genetic divergence 0.2–2.2%) was recovered in *N*. *conoidalis* based on COI; however, two well-supported clades were detected based on ITS1 with the inter-clade genetic divergence of 15.0% (Fig 4; Table 2). Similar phylogenetic inconsistence was detected in *N*. *conoidali*s and *N*. *nodifer* (Figs 2 and 4). The possible mechanism responsible for such phylogenetic inconsistence is the directional introgression [56–57]. Mitochondrial genes fail to detect introgressed haplotypes owing to the maternal inheritance when directional introgression occurs. Indeed, our data partially supported the introgression hypothesis here when haplotypes/alleles were checked based on individuals. Individuals from the same clades defined by COI were assigned into different clades based on ITS1, and vice versa (Figs 2 and 4).

In general, the taxonomy and species identification of *Nassarius* is highly debated and largely disordered, as morphological difference is minor among related species in such a species-rich genus [58]. For example, *N*. *cremates*, *N*. *euglyptus* and *N*. *siquijorensis* are not easy to differentiate based on their morphology [59]. Morphological variation is often detected in specimens collected from different sites, even from the same sites. For instance, we found substantially different shell morphologies and aperture structures in several species such as *N*. *pullus* (S2 Fig) in South China Sea, with a high level of thin and fragile shells and closed inner lips. The use of molecular identification, as well as cross-check with morphology, can largely solve the taxonomic debates in *Nassarius* [18, 22].

The use of subgenus is common in taxonomic description in the species-rich genus of *Nassarius* [60]. Usually, the presence of accessory lateral plates was used to define subgenus [60]. However, the presence of accessory lateral plates was not consistent within subgenera. For example, only a part of species in the defined subgenus of *Zeuxis* has accessory lateral plates [19]. However, the information on accessory lateral plate, such as the presence or not and size, is valuable to differentiate related species. For example, *N*. *hirasei* was considered to be the synonym of *N*. *siquijorensis* [19]; however, the use of the size of accessory lateral plates can easily differentiate these two species, and our phylogenetic analyses confirmed the validity of these two species (Fig 3). Our phylogenetic analyses, together with others [18, 22], did not support the monophyly of the commonly used subgenera (Fig 3). Consequently, the common used division of subgenus in *Nassarius* is not phylogenetically valid, and upgrade of subgenus to genus as suggested by several studies, should be performed with extra cautions.

## Supporting information

S1 Fig Radular teeth in all *Nassarius* mud snail species analyzed in this study. (DOC)

S2 Fig Morphological variation in shell and aperture structures in *Nassarius pullus* detected in the South China Sea. The left figure shows the normal and regular morphology in shell and aperture, while the right one illustrates thin and fragile shells and closed inner lips.
(DOC)

S1 Table *Nassarius* Species identified from the Chinese coast. The species name, collection site, number of individuals analyzed for both genetic markers [mitochondrial cytochrome *c* oxidase subunit I (COI) and nuclear internal transcribed spacer 1 (ITS 1)], number of haplotypes/alleles detected at both genetic markers, and GenBank accession number for each haplotype/allele are shown.
(DOC)
