# Supplementary material for: Phylogeography in Nassarius mud snails: Complex patterns in congeneric species
Source: PLoS One. 2017 Jul 12;12(7):e0180728. doi: 10.1371/journal.pone.0180728 (PMC5507531; doi:10.1371/journal.pone.0180728)

**Article type: Research paper**

**Phylogeography in *Nassarius* mud snails: complex patterns in congeneric species**

Chuanliang Pu^1,2#^, Haitao Li^3#^, Aijia Zhu^3^, Yiyong Chen^1,2^, Yan Zhao^1,2^, Aibin Zhan^1,2*^

^1^ Research Center for Eco-Environmental Sciences, Chinese Academy of Sciences, 18 Shuangqing Road, Haidian District, Beijing 100085, China;

^2^ University of Chinese Academy of Sciences, 19A Yuquan Road, Shijingshan District, Beijing 100049, China;

^3^ South China Sea Environmental Monitoring Center, State Oceanic Administration, 155 Xingang Road West, Guangzhou, Guangdong 510300, China;

^#^ These two authors contribute equally to this work.

*** Corresponding authors:** Dr. Aibin Zhan, Research Center for Eco-Environmental Sciences, Chinese Academy of Sciences, 18 Shuangqing Road, Haidian District, Beijing 100085, China; Email: [zhanaibin@hotmail.com](mailto:zhanaibin@hotmail.com), Phone: (+86)-10-6284-9882, Fax: (+86)-10-6284-9882.

**Fig S1** Radular teeth in all *Nassarius* mud snail species analyzed in this study.


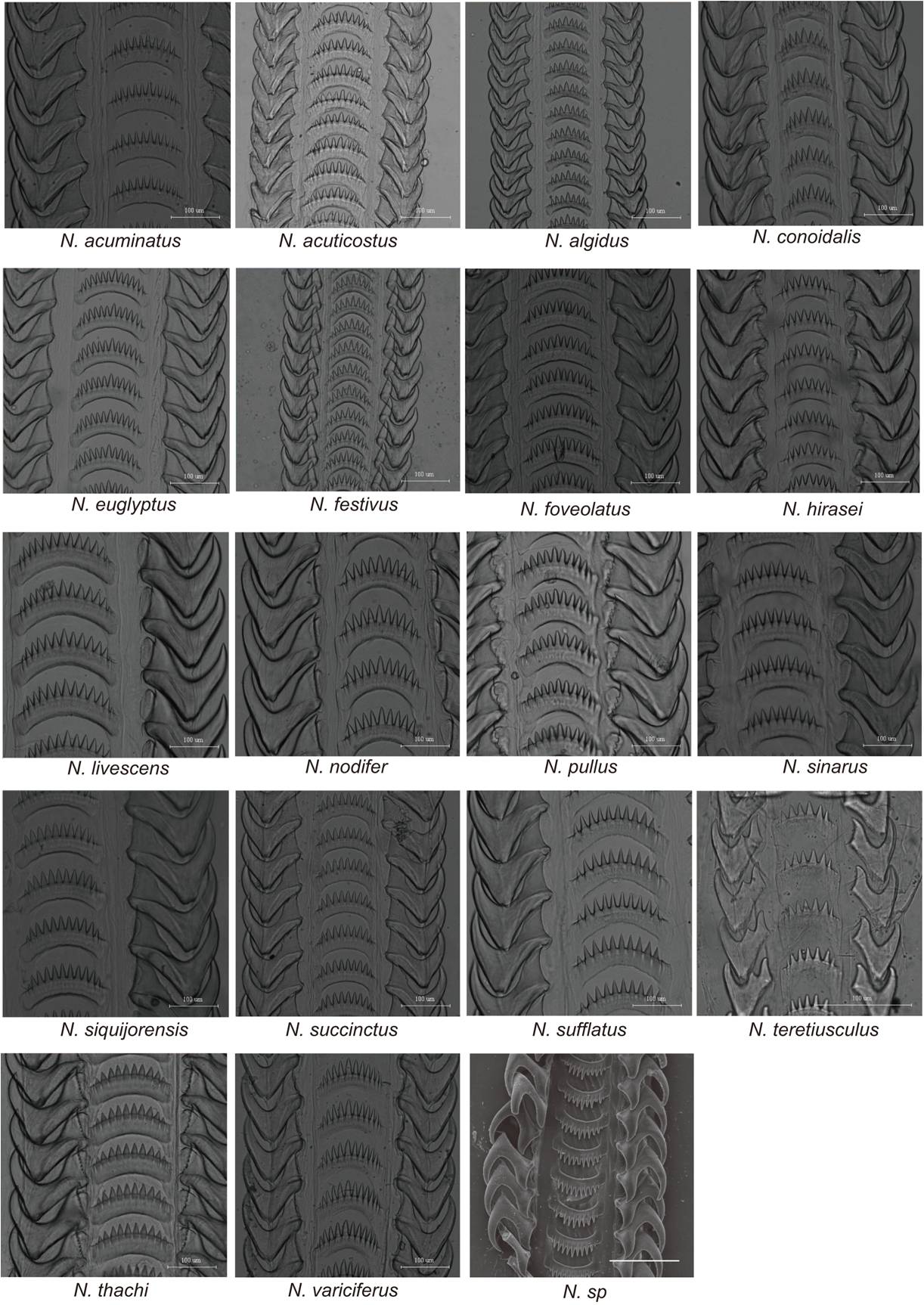

Supplement: S1 Fig — (DOC) [file pone.0180728.s001.doc]
