# Supplementary material for: Phylogeography in Nassarius mud snails: Complex patterns in congeneric species
Source: PLoS One. 2017 Jul 12;12(7):e0180728. doi: 10.1371/journal.pone.0180728 (PMC5507531; doi:10.1371/journal.pone.0180728)

**Article type: Research paper**

**Phylogeography in *Nassarius* mud snails: complex patterns in congeneric species**

Chuanliang Pu^1,2#^, Haitao Li^3#^, Aijia Zhu^3^, Yiyong Chen^1,2^, Yan Zhao^1,2^, Aibin Zhan^1,2*^

^1^ Research Center for Eco-Environmental Sciences, Chinese Academy of Sciences, 18 Shuangqing Road, Haidian District, Beijing 100085, China;

^2^ University of Chinese Academy of Sciences, 19A Yuquan Road, Shijingshan District, Beijing 100049, China;

^3^ South China Sea Environmental Monitoring Center, State Oceanic Administration, 155 Xingang Road West, Guangzhou, Guangdong 510300, China;

^#^ These two authors contribute equally to this work.

*** Corresponding authors:** Dr. Aibin Zhan, Research Center for Eco-Environmental Sciences, Chinese Academy of Sciences, 18 Shuangqing Road, Haidian District, Beijing 100085, China; Email: [zhanaibin@hotmail.com](mailto:zhanaibin@hotmail.com), Phone: (+86)-10-6284-9882, Fax: (+86)-10-6284-9882.

**Fig S2** Morphological variation in shell and aperture structures in *Nassarius pullus* detected in the South China Sea. The left figure shows the normal and regular morphology in shell and aperture, while the right one illustrates thin and fragile shells and closed inner lips.


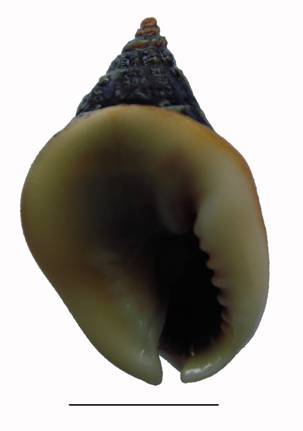

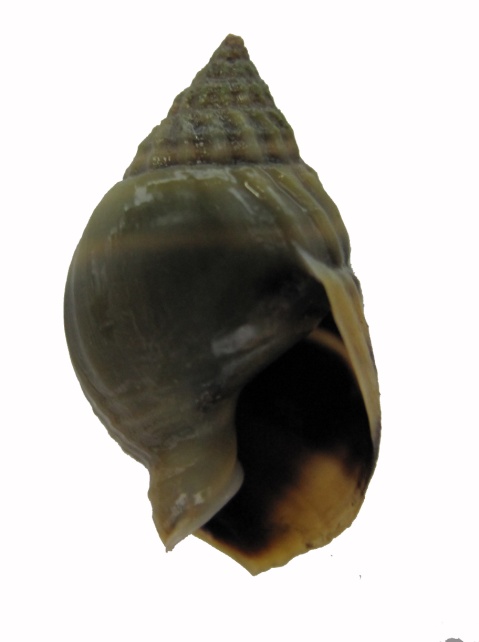

Supplement: S2 Fig — The left figure shows the normal and regular morphology in shell and aperture, while the right one illustrates thin and fragile shells and closed inner lips. (DOC) [file pone.0180728.s002.doc]
