# Supplementary material for: Phylogeography in Nassarius mud snails: Complex patterns in congeneric species
Source: PLoS One. 2017 Jul 12;12(7):e0180728. doi: 10.1371/journal.pone.0180728 (PMC5507531; doi:10.1371/journal.pone.0180728)
Supplement: S1 Table — The species name, collection site, number of individuals analyzed for both genetic markers [mitochondrial cytochrome c oxidase subunit I (COI) and nuclear internal transcribed spacer 1 (ITS 1)], number of haplotypes/alleles detected at both genetic markers, and GenBank accession number for each haplotype/allele are shown. (DOC) [file pone.0180728.s003.doc]

**Article type: Research paper**

**Phylogeography in *Nassarius* mud snails: complex patterns in congeneric species**

Chuanliang Pu^1,2#^, Haitao Li^3#^, Aijia Zhu^3^, Yiyong Chen^1,2^, Yan Zhao^1,2^, Aibin Zhan^1,2*^

^1^ Research Center for Eco-Environmental Sciences, Chinese Academy of Sciences, 18 Shuangqing Road, Haidian District, Beijing 100085, China;

^2^ University of Chinese Academy of Sciences, 19A Yuquan Road, Shijingshan District, Beijing 100049, China;

^3^ South China Sea Environmental Monitoring Center, State Oceanic Administration, 155 Xingang Road West, Guangzhou, Guangdong 510300, China;

^#^ These two authors contribute equally to this work.

*** Corresponding authors:** Dr. Aibin Zhan, Research Center for Eco-Environmental Sciences, Chinese Academy of Sciences, 18 Shuangqing Road, Haidian District, Beijing 100085, China; Email: [zhanaibin@hotmail.com](mailto:zhanaibin@hotmail.com), Phone: (+86)-10-6284-9882, Fax: (+86)-10-6284-9882.

**Table S1** *Nassarius* Species identified from the Chinese coast. The species name, collection site, number of individuals analyzed for both genetic markers [mitochondrial cytochrome *c* oxidase subunit I (COI) and nuclear internal transcribed spacer 1 (ITS 1)], number of haplotypes/alleles detected at both genetic markers, and GenBank accession number for each haplotype/allele are shown.

| **Species** | **Collection sites** | **No. of individuals**  **analyzed for COI** | **No. of**  **haplotypes** | **COI haplotypes/**  **Accession nos.** | **No. of individuals**  **analyzed for ITS1** | **No. of**  **alleles** | **ITS1 alleles/**  **Accession nos.** |
| --- | --- | --- | --- | --- | --- | --- | --- |
| *N. acuminatus* | 4, 22 | 11 | 6 | C_NAc1-6/  KY100528-33 | 4 | 4 | I_NAc1-4/  KY100798-801 |
| *N. acuticostus* | 12, 13 | 2 | 1 | C_NAcu1/  KY100534 | 2 | 2 | I_NAcu1-2/  KY100732-3 |
| *N. algidus* | 9 | 2 | 2 | C_NAl1-2/  KY100535-6 | 1 | 1 | I_NAl1/  KY100820 |
| *N. sp* | 3 | 3 | 2 | C_Nsp1-2/  KY100537-8 | 2 | 2 | I_Nsp1-2/  KY100816-7 |
| *N. conoidalis* | R8, 1, 2, 3, 23, 24 | 26 | 19 | C_NCo1-19/  KY100539-57 | 17 | 17 | I_NCo1-17/  KY100765-81 |
| *N. euglyptus* | 5 | 3 | 3 | C_NEu1-3/  KY100558-60 | 1 | 1 | I_NEu1/  KY100865 |
| *N. festivus* | R1-R6, 11 | 40 | 26 | C_NFe1-26/  KY100561-86 | 34 | 6 | I_NFe1-6/  KY100734-9 |
| *N. foveolatus* | 7 | 1 | 1 | C_NFo1/  KY100587 | 1 | 1 | I_NFo1/  KY100821 |
| *N. hirasei* | 6 | 3 | 2 | C_NHi1-2/  KY100588-9 | 3 | 2 | I_NHi1-2/  KY100818-9 |
| *N. livescens* | R10 | 4 | 3 | C_NLi1-3/  KY100590-2 | 4 | 2 | I_NLi1-2/  KY100796-7 |
| *N. nodifer* | R7, R8, R13, 10 | 58 | 29 | C_NNo1-29/  KY100593-621 | 15 | 14 | I_NNo1-14/  KY100782-95 |
| *N. pullus* | R4, R6, R7, 16 | 10 | 6 | C_NPu1-6/  KY100622-7 | 8 | 8 | I_NPu1-8/  KY100837-44 |
| *N. sinarus* | 8, 20 | 26 | 18 | C_NSin1-26/  KY100628-45 | 26 | 15 | I_NSin1-15/  KY100822-36 |
| *N. siquijorensis* | R8, 1, 2, 18, 21 | 23 | 17 | C_NSiq1-17/  KY100646-62 | 12 | 10 | I_NSiq1-10/  KY100806-15 |
| *N. succinctus* | R7, R9, 18, 20 | 54 | 26 | C_NSuc1-26/  KY100663-88 | 38 | 21 | I_NSuc1-21/  KY100741-61 |
| *N. sufflatus* | 1, 14, 15 | 9 | 6 | C_NSuf1-6/  KY100689-94 | 4 | 3 | I_NSuf1-3/  KY100762-4 |
| *N. teretiusculus* | 17 | 1 | 1 | C_NTe1/  KY100695 | 1 | 1 | I_NTe1/  KY100740 |
| *N. thachi* | 19, 25, 26, 27, 28 | 10 | 7 | C_NTh1-7/  KY100696-702 | 6 | 4 | I_NTh1-4/  KY100802-5 |
| *N. variciferus* | R3, R11, R12, 18 | 44 | 29 | C_NVa1-29/  KY100703-31 | 23 | 20 | I_NVa1-20/  KY100845-64 |
